# NASA Space Flight Human-System Standard: enabling human spaceflight missions by supporting astronaut health, safety, and performance

**DOI:** 10.1038/s41526-023-00275-2

**Published:** 2023-04-03

**Authors:** Sarah D. Childress, Tara C. Williams, David R. Francisco

**Affiliations:** 1 JES Tech, Houston, TX USA; 2 Office of the Chief Health & Medical Officer, NASA Headquarters, Washington, DC USA; 3 KBR, Houston, TX USA

**Keywords:** Risk factors, Scientific community

## Abstract

The purpose of this paper is to describe NASA’s approach to establishing and maintaining a set of Agency-level Space Flight Human System Standards managed by the Office of the Chief Health and Medical Officer (OCHMO) at NASA that enables space flight missions by minimizing health risks to astronauts, providing vehicle design parameters, and supporting the performance of both flight and ground crews. NASA standards capture and provide knowledge, guidelines, thresholds and limits for the successful design and operation of spacecrafts and missions. The NASA Space Flight Human-System Standard (NASA-STD-3001) consists of two separate volumes of technical requirements: NASA-STD-3001 Volume 1: Crew Health addresses the requirements needed to support astronaut health and provide medical care; NASA-STD-3001 Volume 2: Human Factors, Habitability, and Environmental Health addresses human-integrated vehicle system design and operational requirements that will maintain astronaut safety and promote human performance. These standards are managed by an OCHMO team who continuously works with national and international subject matter experts and with each space flight program to provide the best technical requirements and implementation documentation to support the development of new programs. Through partnerships across the space flight industry, these technical requirements are constantly evolving to enable successful implementation of NASA programs and the commercialization of human space flight.

## Introduction

### Hazards of space flight

There are five primary hazards of space flight^1,2^ that NASA-STD-3001^3^ fundamentally seeks to address. These primary hazards include:

1. Radiation Exposure: Outside of Earth’s natural protection, radiation exposure is abundant in space. Crew exposure to the space radiation environment may pose health risks including the risk of neoplasm, damage to the central nervous system, altered cognitive functioning, reduced motor functioning, and acute behavioral changes. In addition, based on analysis and limited data from the International Space Station^4^, the impact of radiation on pharmaceuticals and food should be minimal, but additional research is being conducted to fully assess the impact^5,6^. Sources of increased radiation exposure include galactic cosmic rays, solar particle events, and onboard radioactive technologies. Vehicle design and engineering can provide a certain level of protection to crewmembers from radiation exposure, however not all types of radiation can be shielded from, and crewmembers are invariably exposed to higher levels of radiation during spaceflight compared to on Earth. Reference *Radiation Protection* NASA OCHMO Technical Brief for additional information^7^.
2. Hostile/Closed Environment: Spacefaring humans must depend on an enclosed vehicle to survive, which involves inherent risks due to engineering (i.e., oxygen, pressure, temperature, noise, lighting, carbon dioxide levels, etc.). The habitability of the spacecraft is imperative for astronaut health and safety. The spacecraft must also provide astronauts the essential elements they require to live and work in space, including food, body waste management, sleep accommodations, and exercise. Reference *Acceleration, Acoustics, Artemis Lighting, Cabin Architecture, Carbon Dioxide, Decompression Sickness (DCS), Environmental Control and Life Support System (ECLSS), Food and Nutrition, Electrical Shock, Lighting Design, Sleep Accommodations, Vehicle Hatches, Waste Management*, and *Water* NASA OCHMO Technical Briefs for additional information^8–21^.
3. Isolation and Confinement: Crewmembers live and work within a small, enclosed environment with other crewmembers for extended periods of time during space flight. Even with careful selection of crewmembers and extensive training, interpersonal and behavioral health issues within the crew are expected. Workload considerations, circadian desynchronization and sleep disturbances, and lack of communication with Earth all play a role in potential performance decrements, negative health outcomes, and potentially loss of mission objectives. NASA has developed several methods to monitor crewmembers’ behavioral health status during space flight and developed tools and technology to identify potential issues earlier and provide the appropriate treatment or intervention. Reference *Behavioral Health and Performance, Behavioral Health Mishaps, Cognitive Workload, Usability, Workload, Error, and Sleep Accommodations* NASA OCHMO Technical Briefs for additional information^18,22–25^.
4. Distance from Earth: As the future of space travel moves toward longer-duration missions beyond lower-Earth Orbit to the Lunar surface and Mars, distance from Earth will become one of the biggest obstacles of safe and productive human space flight. Lack of resupply vehicles, communication delays, potential medical emergencies, and equipment failures are just some of the obstacles that astronauts will face during future space flight missions. Reference *Spaceflight Experience and Medical Care Technical Brief*^26^.
5. Altered Gravity Environment: The experience of astronauts transitioning from one gravitational field to another introduces a host of considerations for crew health, safety, and performance. Future missions will involve several changes in the gravity environment, thus operational parameters must implement all strategies available to mitigate these decrements as much as possible. In addition, humans living and working in space for extended periods of time leads to many health complications that makes returning to Earth’s gravitational environment difficult. Changes to bones, muscles, eyes (Spaceflight-Associated Neuro-Ocular Syndrome, or SANS), and the cardiovascular system are just some of the problems that astronauts deal with when returning from space flight. Reference *Principles of Clinical Medicine for Space Flight*^27^ for a comprehensive review of the human systems affected by space flight and microgravity. Appropriate countermeasures and interventions are imperative to keep crewmembers healthy, safe, and performing at their highest potential. Reference *Bone Loss, Spaceflight Experience and Medical Care, and Pharmaceuticals and Medications* NASA OCHMO Technical Briefs for additional information^26,28,29^.

When combined, these five hazards of space flight create a complicated landscape and impact the ability to support the health, safety, and optimal performance of space flight crews. In addition, as mission duration lengths increase with a greater focus on deep space exploration, these hazards become even more important to address in order to enable the future of space travel.

### NASA human system risk mitigation

Over the past 50+ years of human space flight, NASA has collected an evidence base of medical, environmental and research data on the effects of space flight on the human body. However, even with years of collected data, NASA has a relatively small population of flown astronauts, making it difficult to fully understand the effects of space flight hazards. In order to address this difficulty, NASA implemented a new cross-discipline process to understand the hazards of space flight and gather, assess, and correlate evidence to better predict the probability of an event occurring and the level of consequence the event would have on human health and productivity. This strategy provides the ability to weigh the risks relative to each other and leads to effective tradeoffs to ensure maximal mission success with the least risk to human health^3,30^. The Human System Risk Board (HSRB) was created to implement this process by the NASA Health and Medical Technical Authority. Its responsibility includes implementing a consistent process of gathering and assessing evidence, providing a likelihood and consequence of the risks, and in turn providing recommendations to updating the NASA-STD-3001 documents so that the set of technical requirements remains up to date with the most current knowledge.

### Overview of NASA-STD-3001

**Fig. 1:**
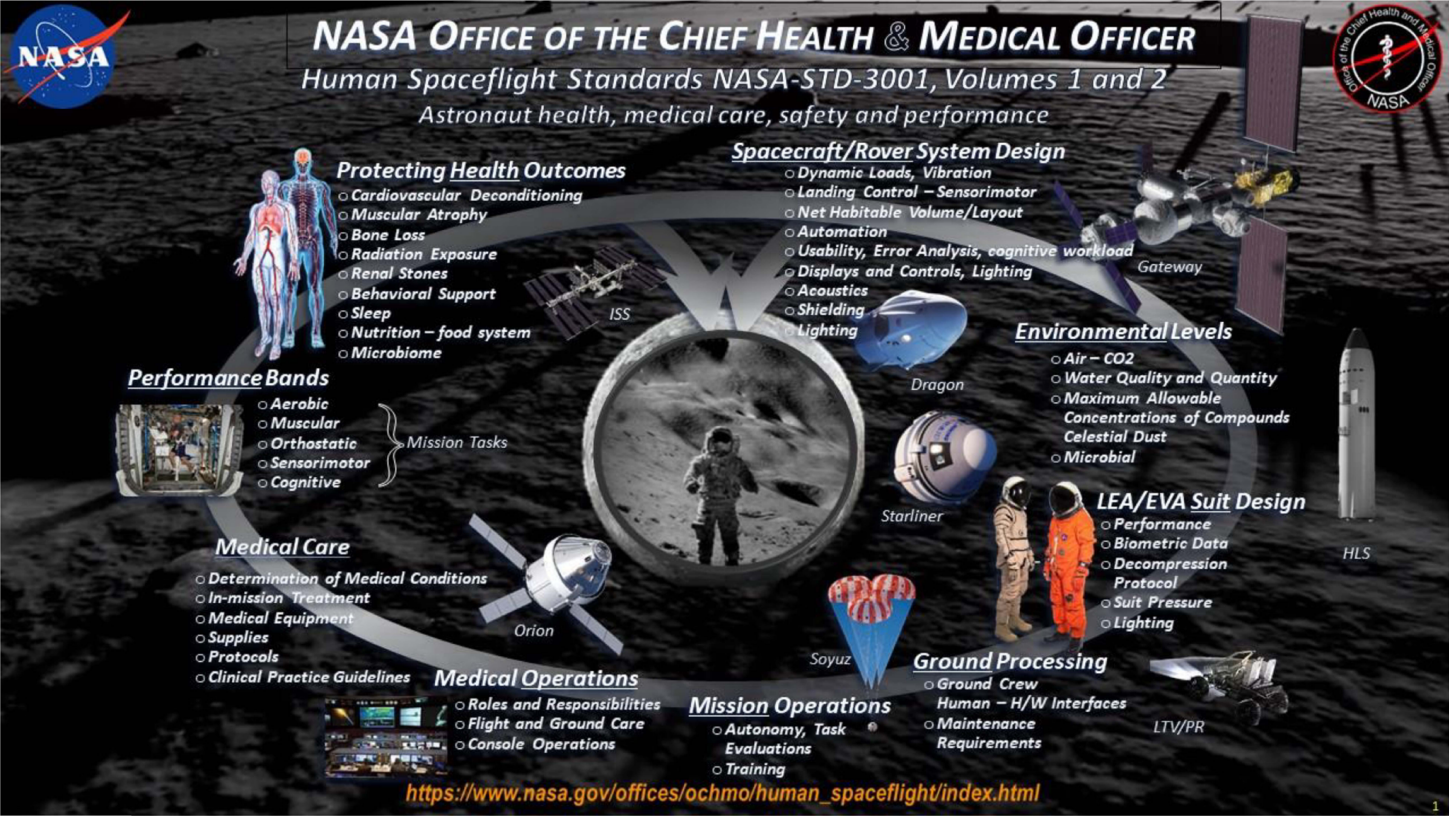
Overview of NASA-STD-3001. An overview of the considerations for astronaut health and performance, and topics addressed in NASA-STD-3001 to guide designs to protect the crew.

The first set of space flight human system standards was baselined Agency-wide in late 1980s and was known as NASA-STD-3000: Man-System Integration Standards (MSIS)^31^. It was first adopted by the International Space Station Program (ISSP) and written primarily for the International Space Station (ISS) infrastructure. As time passed and the development of new programs with needs separate from the ISS emerged, an updated set of standards with broader applicability was needed to replace NASA-STD-3000. Driving reasons behind this update included the fact that the original standard was too comprehensive, was too focused on design solutions, and raised questions on the verifiability of some technical requirements. After a second variation of NASA-STD-3000 and an accompanying handbook were created^32^, NASA-STD-3001 was baselined and implemented in 2009 by the NASA OCHMO. These standards are applicable to all human space flight programs and projects, enabling the creation of human-centered program-specific technical requirements. Each technical requirement within these documents includes a ‘shall’ statement that must be considered in the development of a program-specific set of human-systems design requirements. Technical requirements include rationales which provide additional information and justification for the purpose of the technical requirement, as well as some potential guidance on how to verify the technical requirements being met. Figure 1 provides an overview of the topics addressed by NASA-STD-3001.

### NASA-STD-3001 volume 1, crew health

Volume 1 of NASA-STD-3001 focuses on human physiological functioning and views the human system as an important part of the overall vehicle design process and mission design. It sets the standards for fitness for duty, space permissible exposure limits, permissible outcome limits, medical care, medical diagnosis, intervention, treatment and care, and countermeasures. Aerospace medicine is deeply rooted in preventive care in addition to being prepared to respond to known physiological and psychological challenges of space flight, as well as unexpected injury and illnesses that could impact crewmembers during their career as an astronaut. Volume 1 seeks to address this comprehensive approach through screening, preventive healthcare strategies, medical care, launch and landing contingencies, and post-mission care, reconditioning, and long-term monitoring. NASA-STD-3001 Volume 1 contains 72 technical requirements as of Revision B published in 2022 that are levied against all human-integrated space flight programs. Table 1 provides a sample of technical requirements from NASA-STD-3001 Volume 1.

**Table 1:** Selected examples of technical requirements from NASA-STD-3001 volume 1.

Section and standard title	Shall statement	Rationale
3.1.8 In-Mission Evacuation to Definitive Medical Care Facilities	[V1 3008] Plans *shall* be available to transport severely ill or injured crewmember(s) to appropriate Medical Care Facilities, including Definitive Medical Care Facilities (DMCF) in the event of a contingency.	*[Rationale: If a return to Earth of a severely ill or injured crewmember is possible and is undertaken, coordination with suitable DMCFs in proximity to potential landing sites will be made in advance of the crewmember’s landing to ascertain readiness of the facility to accept and implement immediate medical care. Mobile ground resources with the capability to initiate medical care en route to the DMCF will be deployed at potential landing sites.]*
4.8.1 As Low as Reasonably Achievable (ALARA) Principle	[V1 4029] All crew radiation exposures *shall* be minimized using the ALARA principle.	*[Rationale: It is important to minimize crew health risk due to radiation exposure by decreasing crew radiation exposure from all sources using the ALARA principle. The ALARA principle is a fundamental guiding principle for radiation protection which requires programs to minimize radiation exposures below the limits/standards within the design constraints of the mission.]*
6.2.1 Private Medical Communication (PMC) Schedule	[V1 6002] A PMC *shall* be scheduled on a routine basis, as determined by the Flight Surgeon, at a frequency dictated for short- or long-duration missions.	*[Rationale: Real time communications are preferred for all PMCs; however, when missions have communication delays, different modalities can be considered (*e.g.*, stored/ forward communications).]*

### NASA-STD-3001 volume 2, human factors, habitability, and environmental health

Volume 2 of NASA-STD-3001 focuses on human-systems integration, including human physical and cognitive capabilities and limitations, and defines requirements for spacecrafts (including orbiters, habitats, and suits), internal environments, ground processing, facilities, payloads, and related requirement, hardware, and software systems. These include requirements to the design of systems that directly interface with the flight crew such as environmental support systems, architecture, controls and displays, and operations, as well as requirements for the design of systems that both ground and/or flight crew access during assembly, test, checkout, or troubleshooting procedures supporting ground processing, launch, landing, and recovery operations. NASA-STD-3001 Volume 2 contains 471 technical requirements as of Revision C published in 2022 that are levied against all human space systems and must be tailored for each individual system to ensure that the end product meets all the requirements. Table 2 provides a sample of technical requirements from NASA-STD-3001 Volume 2.

**Table 2:** Selected Examples of Technical Requirements from NASA-STD-3001 Volume 2.

Section and standard title	Shall statement	Rationale
4.1.3.1 Crew Operational Loads	[V2 4104] The system *shall* be operable by crew during all phases of flight, including prelaunch, ascent, orbit, entry, and postlanding, with the lowest anticipated strength as defined in Appendix F, Physical Characteristics and Capabilities, Section F.7.	*[Rationale: All crewmembers need to be able to perform any planned tasks efficiently and effectively. The crew operating load data in Appendix F defines the lowest anticipated forces that can be applied by crewmembers in unsuited, suited-unpressurized, and suited-pressurized conditions, taking into account deconditioning and factors of safety for critical tasks. A task analysis that identifies planned crew tasks, hardware interfaces, expected postures, and task criticality, frequency, and duration is used with Appendix F to define the maximum acceptable value for actuation and continued operation of hardware interfaces. Guidance on the evaluation of design using crewmember strength data can be found in NASA/TP-2014-218556 Human Integration Design Process (HIDP). The intent of this requirement is to accommodate the entire potential user population, not just meet the criteria in the datasets provided, which provide the most frequently used values. Identification of postures or forces not provided in the table needs coordination and concurrence from NASA Stakeholders. A tailored data set may be provided by NASA based on program or mission specific criteria.]*
6.2.9.3 Lunar Dust Contamination	[V2 6053] The system *shall* limit the levels of lunar dust particles <10 μm in size in the habitable atmosphere below a time-weighted average of 0.3 mg/m3 during intermittent daily exposure periods that may persist up to 6 months in duration.	*[Rationale: This limit was based on detailed peer-reviewed studies completed by the Lunar Atmosphere Dust Toxicity Assessment Group (LADTAG) and is specific to the conditions relevant to the lunar surface,* i.e.*, this requirement would not necessarily be applicable to other missions. The requirement assumes that the exposure period is episodic and is limited to the time before ECLSS can remove the particles from the internal atmosphere (assumed as 8* *h post introduction). Although the requirement is being conservatively applied to all inhalable particles (all particles* *≤* *10* *μm), it is most applicable to dusts in the respirable range (≤2.5* *μm) that can deposit more deeply into the lungs. Studies show that the particle size of lunar dust generally falls within a range of 0.02–5* *µm. The ability to meet this requirement will depend upon factors such as the level of lunar dust introduction and ECLSS removal rates. The monitoring of dust is captured in [V2 6153] Celestial Dust Monitoring and Alerting.]*
10.6.1 Automated and Robotic System Provision	[V2 10100] Automated or robotic systems *shall* be provided when crew cannot reliably, safely, or efficiently perform assigned tasks.	*[Rationale: Systems are to have automated or robotic solutions that can perform tasks where (1) crew cannot respond as quickly, precisely, or repeatedly as necessary; (2) crew cannot physically complete the task; or (3) using automation/robotic solutions reduces crew risk exposure (*e.g.*, high radiation environments, limited life support availability).]*

### Creation of program requirements from NASA-STD-3001

**Fig. 2:**
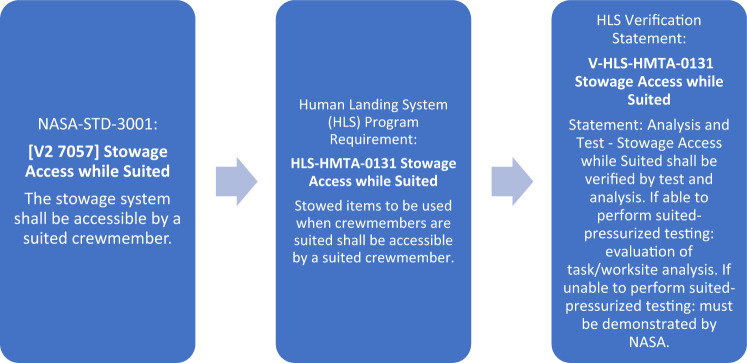
Example of NASA-STD-3001 to program requirement flow. An example of how programs tailor individual technical requirements from NASA-STD-3001 to create and verify program-specific requirements.

Each individual NASA program that will include human-rated systems must implement NASA-STD-3001 in the development of their specific program requirements. The program is responsible for reviewing the set of standards, identifying the technical requirements that are applicable to their specific program/design reference mission, and tailoring the standards into detailed requirements to be implemented in the program. In addition, the programs must provide verification information which details the program’s plan to verify that their requirements are meeting their intent. Examples of a tailored 3001 technical requirement to program requirement and its related verification language is provided in Fig. 2.

### Human space flight standards hierarchy pyramid

**Fig. 3:**
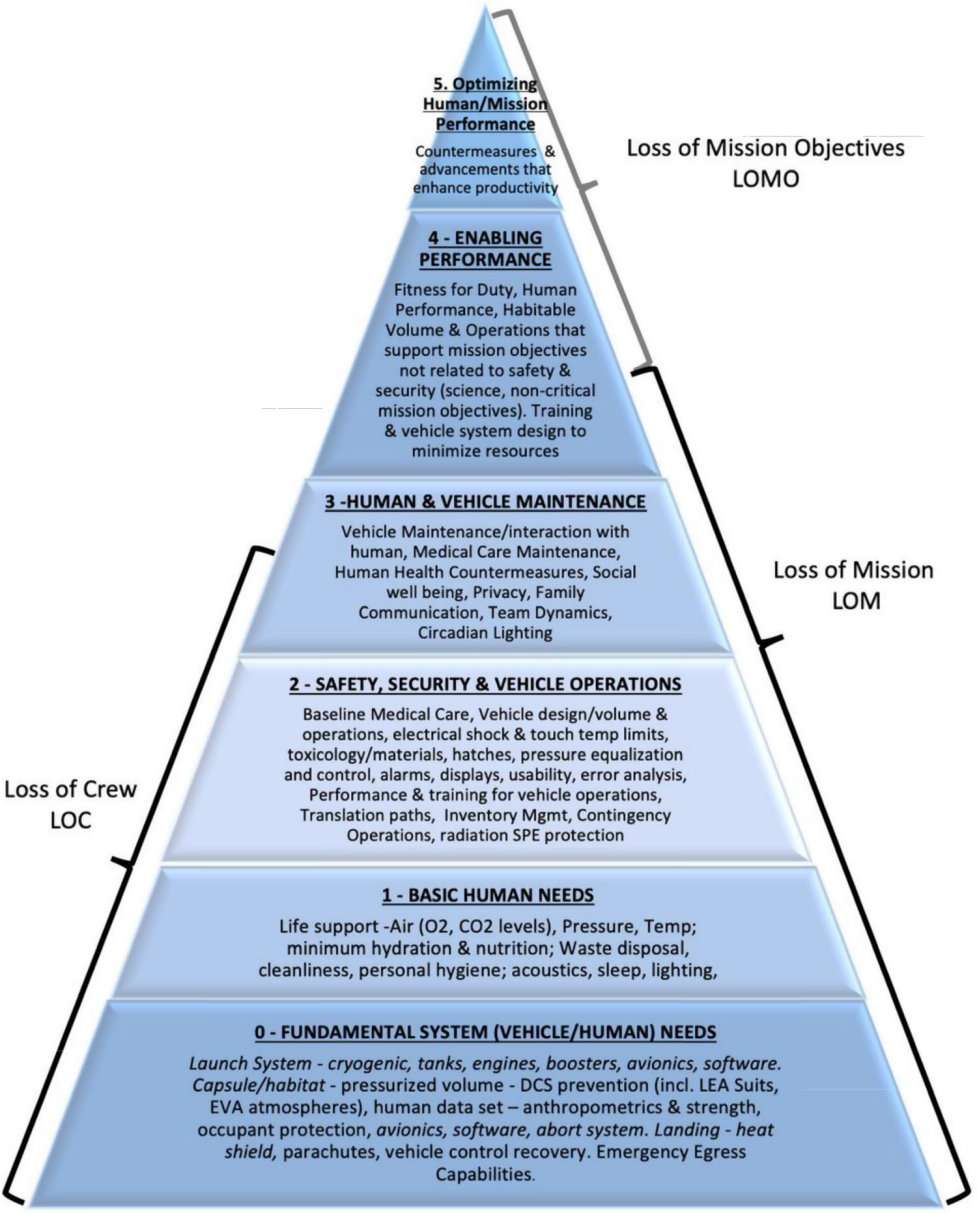
Human space flight standards hierarchy pyramid. The purpose of this pyramid is to provide a top-level view of technical requirements and how each subsection impacts a mission’s success.

During the early formulation stage of a program, it is helpful to assess the cross-relationship of standards and how they impact specific missions and programs. The Human Space Flight Standards Hierarchy (Fig. 3)^33^ was developed to provide a tool to determine how individual technical requirements impact future missions and programs. It provides the ability to look “across” all of the technical requirements and assess their impact on a mission’s success in relation to loss of crew, loss of mission, and loss of mission objectives. The pyramid is broken down into six levels, with the base representing the most fundamental system needs (both vehicle and human) and the top-level representing optimization of human and mission performance. This hierarchy is useful in determining the applicability of technical requirements for programs during the formulation stage of development. The pyramid also helps to categorize technical requirements that increase the probability of achieving mission objectives.

### Human integration design handbook

The Human Integration Design Handbook (HIDH)^32^ is a companion document designed to accompany NASA-STD-3001 Volume 2. It includes information specific to human space flight history and lessons learned, design information, reference data and background information, and application guidance for the wide variety of disciplines and topic areas covered by NASA-STD-3001. The main purpose of the HIDH is to help requirements writers prepare program-specific human requirements and to help designers develop designs and operations for human interfaces in spacecrafts. The scope of the handbook encompasses all crew operations both inside and outside spacecrafts in space and on Lunar or planetary surfaces. It includes design guidelines for crew interfaces with workstations, architecture, habitation facilities, extravehicular activity (EVA) systems; information describing crew human capabilities and limitations (including both physical and cognitive); and environmental support parameters. The potential users of the HIDH include program managers, program/system requirements writers, human factors practitioners, engineers and designers, crews and mission/flight controllers, and training and operations developers.

### Human space flight technical briefs

The OCHMO 3001 Standards Team has developed a repository of human space flight technical briefs^34^ that cover a range of topics. Each technical brief includes information regarding technical data, background, reference information, and application notes to aid with the development of hardware, systems, and vehicles, as well as human needs and limitations. This information is used by vehicle developers, program requirement writers, and medical professionals. These technical briefs integrate content from multiple technical requirements and provide a quick, informative resource to reference when working with NASA-STD-3001. Currently, there are 31 published human space flight technical briefs with additional topics being added on a regular basis. Stakeholders, NASA SMEs, and other potential users are encouraged to submit their ideas for future technical brief topics that would be informative and useful for ongoing work.

### Process for updating NASA-STD-3001

**Fig. 4:**
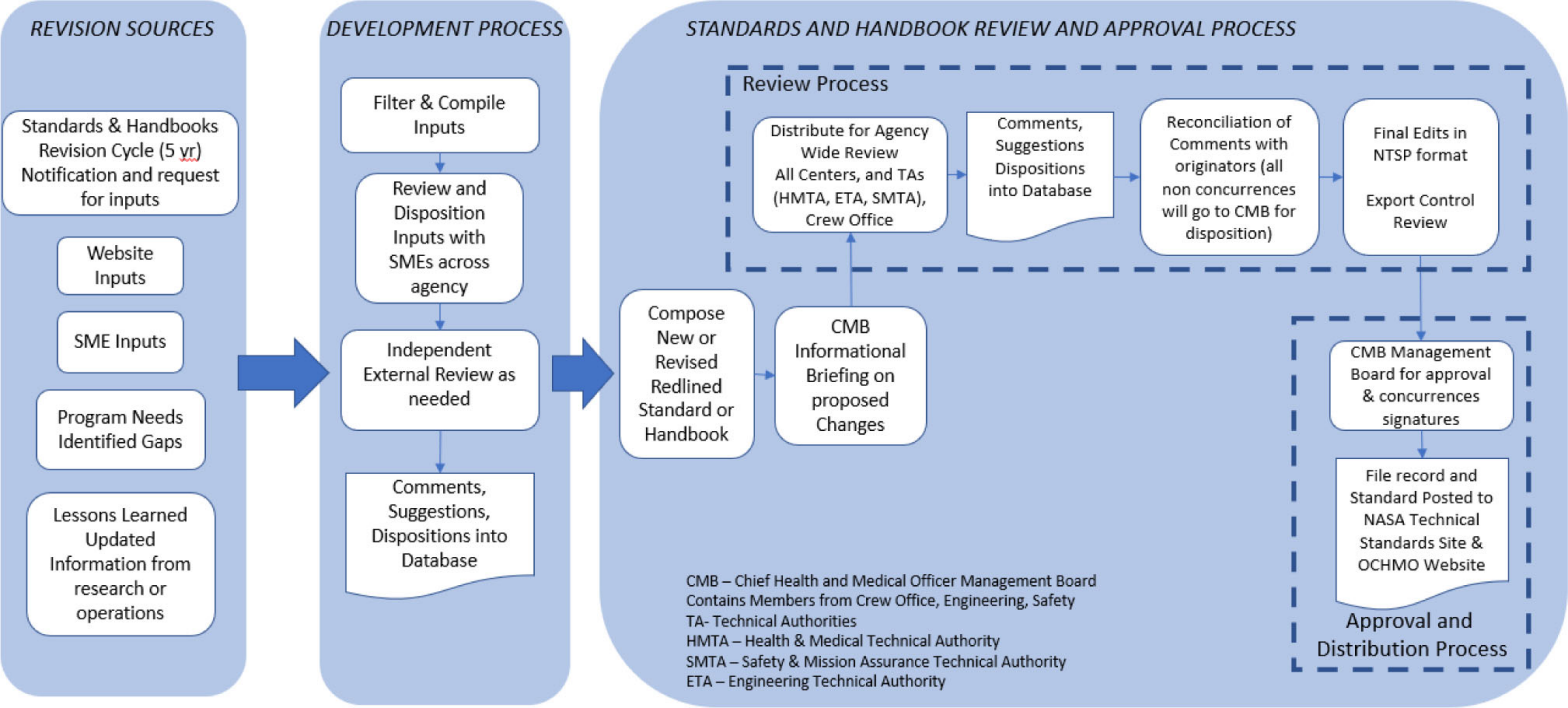
Development and revision process for OCHMO standards and handbooks. The purpose of this graphic is to give readers a better understanding of the process used to revise and approve NASA-STD-3001 and associated documents.

The NASA-STD-3001 documents are reviewed and updated in a continuous cycle in order to keep the technical requirements current and up-to-date with the latest knowledge and guidance. Potential updates and recommendations for the technical requirements are identified through several different avenues, which include but are not limited to:

1. NASA Subject Matter Experts (SMEs) are invaluable resources for ensuring that current technical requirements include relevant and important content for each individual area of interest, utilizing the expertize and experience of the SMEs to provide the best available information to form new and update existing technical requirements.
2. Human System Risk Board (HSRB) as described previously, helps to inform the standards through regular reviews of identified space flight human health risks which includes an overview of the related technical requirements and potential recommendations to update those technical requirements to address any changes or concerns regarding each specific risk.
3. NASA Human Research Program (HRP) is a program dedicated to conducting ongoing space flight research in many facets of human space travel and includes five elements: the International Space Station Medical Projects, Space Radiation, Human Health Countermeasures, Exploration Medical Capability, and Human Factors and Behavioral Performance^35^. The results from HRP’s research and findings helps to inform the technical requirements and provide important updates using new and updated knowledge,

The process for updating the NASA-STD-3001 documents and related handbooks includes a specific set of steps to be followed to ensure consistent and accurately documented changes to the standards. Figure 4 below provides an overview of this process.

### Future exploration and commercialization of space flight

The future of NASA and human space flight is undergoing significant changes, with the landscape of space travel and exploration turning toward longer-duration missions on the Lunar surface and eventual deep space to Mars. In addition, commercial partners have become the primary contributors to supporting ongoing human space travel and thus it is imperative to consider their role in the future of space flight. The NASA-STD-3001 documents are invaluable resources for the evolving space flight environment, providing commercial partners with the necessary knowledge and guidance to enable safe and effective human space travel. In addition, lessons learned and feedback from previous programs development activities and operations helps to inform future programs on how to appropriately integrate the 3001 technical requirements to achieve their goals and objectives and overall mission success.
